# Cardiac sensitivity to rewards in cognitively inflexible nonclinical participants

**DOI:** 10.7717/peerj.15318

**Published:** 2023-05-08

**Authors:** José Luis Mata, Azahara Leonor Miranda Gálvez, Francisca López Torrecillas, Laura Miccoli

**Affiliations:** 1 Department of Personality, Evaluation and Psychological Treatment, University of Granada, Granada, Andalucía, Spain; 2 Faculty of Humanities and Education Sciences, University of Jaén, Jaén, Andalucía, Spain

**Keywords:** Emotion, Individual differences, Heart rate, Iowa Gambling Task, Psychopathology, Cognitive inflexibility, Compulsivity, Reinforcement learning, Reward sensitivity

## Abstract

**Background:**

In psychopathologies characterized by compulsive decision-making, core impairments include cognitive inflexibility and excessive sensitivity to rewards. It has been posited that traits shared by nonclinical individuals and psychiatric patients could help explain the pathogenesis of compulsive decision-making.

**Methods:**

To investigate whether cognitive inflexibility predisposes nonclinical individuals to poor choices and hyper-reactivity to reward, we recruited people with high and low scores for cognitive persistence and used the Iowa Gambling Task to assess decision-making and cardiac reactivity to monetary gains/losses.

**Results:**

As is frequently observed in psychophysiological research, the data indicated discrepancies among self-reports, behavior, and physiology. Cognitive inflexibility was not related to worse performance; however, monetary gains, in line with the literature, prompted marked cardiac accelerations. Consistent with our research goal, only inflexible participants showed large cardiac accelerations during the largest monetary wins.

**Discussion:**

Taken together, the data confirm an association between cognitive persistence and physiological reward sensitivity in a nonclinical population. The findings are in line with recent theories on the development of compulsive behaviors that consider cognitive inflexibility as a transdiagnostic impairment and predisposing factor for excessive reactivity to rewards, and might act both as a preexisting individual trait and drug-induced deficit.

## Introduction

Theories on impaired decision-making across a range of psychopathologies characterized by compulsivity, including behavioral and substance use disorders, identify cognitive inflexibility as a core transdiagnostic impairment (Parnaudeau, Bolkan & Kellendonk, 2018; Voon et al., 2017; Wyckmans et al., 2019). Cognitive inflexibility broadly refers to difficulty in readjusting one’s choices after changes in reward contingency (Perandrés-Gómez et al., 2021). It is taken to indicate the inability to modify cognitive representations, and hence behavior, in response to changing conditions: the solutions that were appropriate during training are inappropriately applied to new problems (Taatgen et al., 2008). The consequence of such cognitive rigidity, “the tendency of an individual *not* to change” (Schultz & Searleman, 2002), is that the individual perseveres with a behavior despite its negative outcomes (Bechara et al., 1997; Rivalan, Ahmed & Dellu-Hagedorn, 2009). Recent human and animal models (Groman et al., 2019; Wyckmans et al., 2019) suggest that adaptive decision-making alternates efficiently between strategies that focus on long-term benefits and those that focus on immediate gain: long-term strategies are assumed to be deficient in compulsive disorders (Bell & Polaschek, 2017; Voon et al., 2017). However, recent studies hint at a more complex and heterogeneous pattern of alternation between decisional strategies (Collins & Cockburn, 2020; Deserno & Hauser, 2020; Gueguen, Schweitzer & Konova, 2021) and suggest that individual differences play a central role in addiction development (Fraser & Janak, 2019; Groman et al., 2019; Sebold et al., 2017; Vandaele & Ahmed, 2021). An individual trait that seems particularly crucial during the initial phases of reinforcement learning is hypersensitivity to reward, *i.e*., the tendency to overreact to pleasant cues (Fraser & Janak, 2019; Rivalan, Ahmed & Dellu-Hagedorn, 2009; Voon et al., 2017).

Overall, the findings indicate that pathological decision-making tends to be characterized by cognitive inflexibility as well as hyperreactivity to rewards (Banca, Harrison & Voon, 2016; Bell & Polaschek, 2017; Groman et al., 2019; Parnaudeau, Bolkan & Kellendonk, 2018; Perandrés-Gómez et al., 2021; Vandaele & Ahmed, 2021; Voon et al., 2017; Wyckmans et al., 2019). It has been proposed that research on traits that are shared by psychiatric patients and nonclinical individuals might aid understanding the pathogenesis of compulsive disorders (Rivalan, Ahmed & Dellu-Hagedorn, 2009). Accordingly, the present study aimed to answer the following research questions: If participants who are nonclinical exhibit trait cognitive inflexibility, will they show–akin to clinical populations–physiological hyperreactivity to rewards following feedback about wins? Moreover, from a behavioral point of view, will nonclinical participants high in cognitive inflexibility–and therefore potentially prone to maladaptive decisions–display impaired decision-making? Thus, we focused on individual differences in nonclinical participants to identify traits that might contribute to the development of pathological reinforcement learning. The observation of hyperreactivity to rewards and/or impaired decision-making among participants who are nonclinical yet score highly for cognitive inflexibility would further support the role that cognitive inflexibility plays in promoting maladaptive reactivity to rewarding cues.

The idea that physiological reactivity might play a central role in decision-making emerged with the ‘somatic marker’ hypothesis (Bechara et al., 1994; Bechara, Dolan & Hindes, 2002; Bechara & Damasio, 2000), which proposes that, in ambiguous situations, physiological responses associated with certain earlier cues might guide decision-making. The Iowa Gambling Task (IGT) (ibidem) provided a framework to investigate complex decision-making in patients and controls (Bull, Tippett & Addis, 2015; Steingroever et al., 2013). In the IGT, participants try to win as much money as possible by choosing from among four card decks (A–D) 100 times; following each deck choice, they receive feedback on monetary wins or losses. However, the difference between “advantageous” (C, D) and “disadvantageous” decks (A, B) is not intuitive and it has been estimated that, after completing the IGT, about one third of healthy controls still do not understand its underlying logic and perseveres with “disadvantageous” decks despite considerable losses, see also the ‘prominent deck B’ phenomenon (Crone et al., 2004; Rivalan, Ahmed & Dellu-Hagedorn, 2009; Visagan, Xiang & Lamar, 2012).

While performance on the IGT (number of correct trials across successive blocks) is typically characterized by a learning curve and progressively stronger preference for advantageous decks (Crone et al., 2004; Werner, Duschek & Schandry, 2009), the physiological activity that precedes deck choice (typically the skin conductance response (SCR) and heart rate (HR)) suggests that, halfway through the task, disadvantageous decks prompt changes in SCR *before* feedback about losses/wins (Bechara et al., 1996; Goudriaan et al., 2006; Werner, Duschek & Schandry, 2009). Thus, according to the somatic marker hypothesis, anticipatory physiology promotes task understanding (Bechara et al., 1994; Werner, Duschek & Schandry, 2009). In addition, throughout the IGT, *after* each deck choice, feedback about monetary losses/wins allows to investigate the impact of rewarding/punishing cues on post-choice physiology. Here, skin conductance shows less consistent patterns (see Simonovic et al., 2019), whereas HR differentiates rewarding from aversive cues so that feedback on monetary wins, relative to losses, prompt repeated cardiac accelerations (Goudriaan et al., 2006; Herman, Esposito & Tsakiris, 2021; Studer, Scheibehenne & Clark, 2016). In tasks related and unrelated to gambling, cardiac accelerations are observed during rewarding, pleasant cues to such an extent that increasing heart rate during emotionally relevant cues is regarded as a reliable index of appetitive motivation (Bradley, Cuthbert & Lang, 1993; Bradley et al., 2001). In contrast to anticipatory physiology, which is more obvious in good performers, participants seem to react to wins and losses regardless of their level of task understanding (Crone et al., 2004; Rivalan, Ahmed & Dellu-Hagedorn, 2009), which suggests independence of reward sensitivity from the processing of task-relevant information (Rivalan, Ahmed & Dellu-Hagedorn, 2009).

Against this background, in the present study we used the Persistence Scale of the TCI-R (Temperament and Character Inventory-Revisited; Gutiérrez-Zotes et al., 2004) to identify participants scoring high or low on cognitive inflexibility. Consistent with current hypotheses on the role of inflexibility (Groman et al., 2019; Wyckmans et al., 2019) and reward sensitivity (*e.g*., Rivalan, Ahmed & Dellu-Hagedorn, 2009; Studer, Scheibehenne & Clark, 2016; Voon et al., 2017) in the development of addictions, we aimed to investigate: (1) whether participants scoring high in cognitive inflexibility show worse decision-making, *i.e*., more disadvantageous choices, than cognitively flexible participants; (2) whether cognitively inflexible participants show greater cardiac sensitivity after IGT wins; and (3) whether the highest monetary wins amplify individual differences in physiological sensitivity to rewards, which overall would point toward increased physiological reactivity to rewarding, pleasant cues.

The mutual presence of cognitive inflexibility and heightened sensitivity to reward in a sample of nonclinical individuals would further support the hypothesis that they both might contribute to the development of maladaptive reinforcement learning.

## Materials and Methods

### Participants

**Figure 1:**
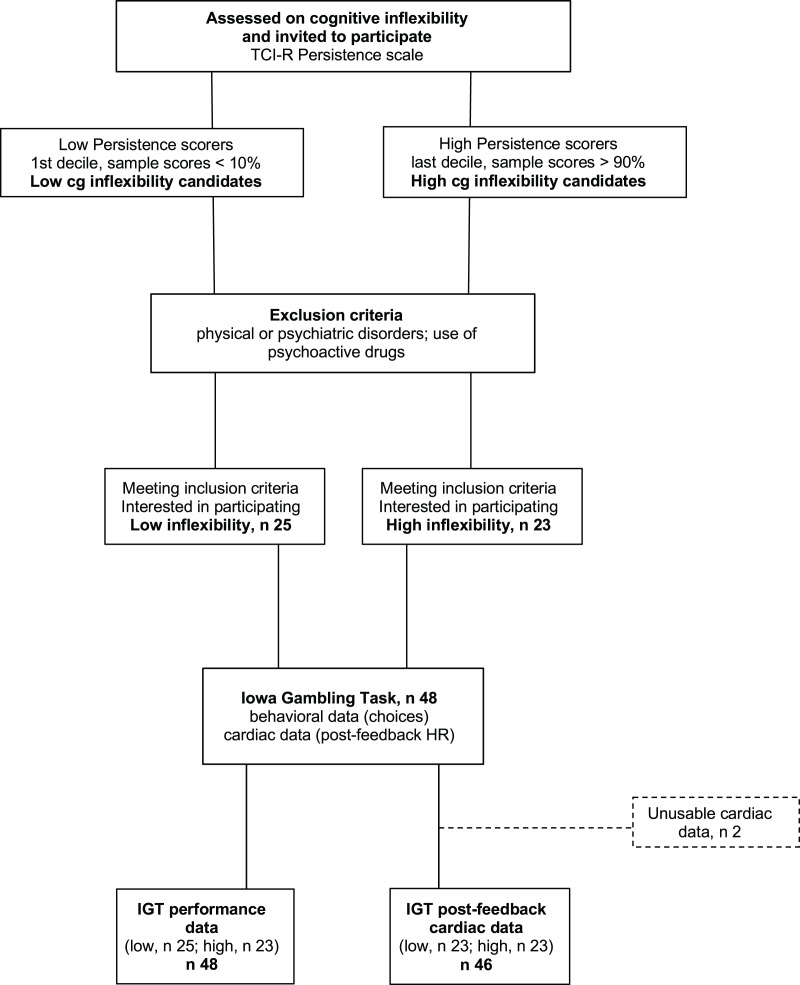
Participant flowchart.

The participants were 48 students (14 men) from the University of Granada, ranging in age from 18 to 37 years (M = 20.6; SD = 4.0) who participated for course credits. Students who did not want to (or could not) participate in the study were offered alternative ways to obtain course credits. The experimenter in charge of data collection was not part of the teaching staff and selected participants based solely on their extreme scores on the TCI-R Persistence subscale, Spanish version (Temperament and Character Inventory-Revisited, Gutiérrez-Zotes et al., 2004). Participant selection was based on a blinded procedure: the experimenter used random digits rather than full names to identify the participants with the most extreme Cognitive Inflexibility scores. From among the initial sample of students who filled out the questionnaire, those with the highest scores (within the top 10% for the whole sample) and lowest scores (within the bottom 10%), *i.e*., participants in the upper (high cognitive inflexibility, M = 125.2, SD = 10.7, *n* = 23) and lower deciles (low cognitive inflexibility, M = 101.4, SD = 7.3, *n* = 25), were selected. Figure 1 shows the study procedure. The Cognitive Inflexibility scores for the upper and lower deciles matched those classified as ‘high’ (M = 124, averaged by sex) and ‘low’ (M = 102, averaged by sex), respectively, based on Spanish normative data (ibidem). Participants were included only if they did not suffer from physical or psychiatric disorders and did not use any psychoactive drugs. Because of equipment failures and/or excessive artifacts, the final sample size was 48 for performance data and 46 for heart rate data. Using G* Power for ANOVA designs (Faul et al., 2007) and including one 2-level within variable and one 2-level between variable, we set error probability ( $\alpha$) at 0.05 and statistical power (1 − $\beta$) at 0.8. Applying these parameters, the current sample size (*n* = 46) was deemed sufficient to detect small effects (partial $\rm \eta$^2^ = 0.02). All participants provided written informed consent. The UGR Ethical Committee approved the experimental protocol (IRB·#2994/CEIH/2022), that complied with the APA ethical standards and the Declaration of Helsinki.

### Instruments

TCI-R Persistence subscale. To assess cognitive inflexibility, the Persistence subscale from the Temperament and Character Inventory-Revisited was administered (Cloninger, 1999; validated Spanish version, Gutiérrez-Zotes et al., 2004). The authors have permission to use this instrument from the copyright holders. The TCI-R Persistence scale consists of 35 5-point Likert items that evaluate Persistence/Cognitive Inflexibility, which is one of the main components of Cloninger’s psychobiological model of personality (ibidem); in the present sample, the scale showed moderate internal consistency (α = 0.73), similar to Spanish normative data (α = 0.76).

Iowa Gambling Task. All participants performed a computerized version of the IGT (Bechara et al., 1994). During 100 trials, participants tried to win as much money as possible by choosing from among four card decks (A–D) with different win and loss contingencies: decks A and B lead to big wins but even bigger losses (disadvantageous decks), whereas decks C and D lead to smaller wins but, in the long term, result in larger net gains (advantageous decks).

### Procedure

Performing the Iowa Gambling Task (IGT), each trial began with all decks displayed on the screen. Immediately after the participant’s choice (left mouse button), if the deck was associated with monetary losses, gains were displayed for 2 s followed by larger losses, shown for 2 s and a variable (1–3 s) intertrial interval (ITI). However, if the deck was associated with monetary gains, these were displayed for 2 s and immediately followed by a 3–5 s ITI. To provide constant feedback, cumulative gains were always in view. At the end of the task, participants were asked if they thought one deck was more advantageous than the others and, if they did, they identified it.

For behavioral data analysis, the IGT is traditionally divided into five blocks of 20 trials, and performance is indexed by the number of advantageous/disadvantageous choices across blocks (*e.g*., Crone et al., 2004); this allows the IGT learning curve to be observed, where participants initially choose decks A and B, but gradually learn that C and D are preferable.

ECG was continuously monitored during a 10-min pre-task rest (baseline), and during the IGT. Nonpolarizable Ag-AgCl electrodes were attached using Eithoven’s lead II (right mid-clavicle, left ankle and right ankle, ground). ECG activity was digitalized at a 1,000 Hz sampling rate using the Biopac MP 100 device (Biopac Systems, Inc., Goleta, CA, USA). Automatic R-wave detection and artifact correction were performed with ECGlab Matlab software (Carvalho et al., 2002) before the extraction of HR values. Mean HR was calculated across baseline and the IGT using KARDIA Matlab software (Perakakis et al., 2010) and custom Matlab scripts (Matlab 2013a; MathWorks Inc., Natick, MA, USA). Monetary feedback was delivered immediately after the deck choice. To examine how post-choice monetary feedback modulated cardiac responses, HR changes were assessed every half-second for 6 s (12 bins) with respect to a 1 s pre-feedback baseline. Zero gain trials (*n* = 48; 1.05% of all trials), which provided no gain or loss, were excluded from the analysis.

### Data analysis

For behavioral data, in line with the literature (Bechara & Damasio, 2000; Werner, Duschek & Schandry, 2009) we examined the number of trials across successive blocks in which cognitively inflexible or flexible participants chose advantageous or disadvantageous decks. A mixed-design analysis of variance of the average number of trials was performed, with Deck Type (two levels: disadvantageous *vs*. advantageous) and Block (five levels: 1–5) as within-participant variables and Cognitive Inflexibility (two levels: low *vs*. high) as a between-participant variable.

For cardiac data, a mixed ANOVA of average HR changes included Monetary Feedback (two levels: losses *vs*. wins) and Bins across each trial (12 levels: 1–6 s) as within-participant variables, and Cognitive Inflexibility (two levels: low *vs*. high) as a between-participant variable. Subsequently, to investigate whether the largest losses or gains prompted distinct cardiac patterns in cognitively inflexible and flexible participants, we examined HR changes after monetary feedback below or above the 10^th^ percentile. An additional mixed ANOVA examined HR changes following the Largest Monetary Feedback (two levels: greatest losses *vs*. greatest wins; repeated-measures variable), while the other variables, *i.e*., Bins and Cognitive Inflexibility, remained unchanged.

All statistical analyses were performed using Statistica v.13 (Dell Inc., Round Rock, TX, USA, 2015). The level of significance was set at 0.05; Greenhouse–Geisser adjustment was applied as necessary, and partial η^2^ was used as a measure of effect size. Based on existing literature, the number of trials was expected to increase across blocks for advantageous decks and monetary gains were expected to prompt larger cardiac accelerations than losses. Accordingly, *a priori* pairwise comparisons were planned and performed to test specific contrasts. All graphs include 95% CIs, which display the variability around the mean more accurately and clearly than standard errors and standard deviations (Motulsky, 2010).

## Results

**Figure 2:**
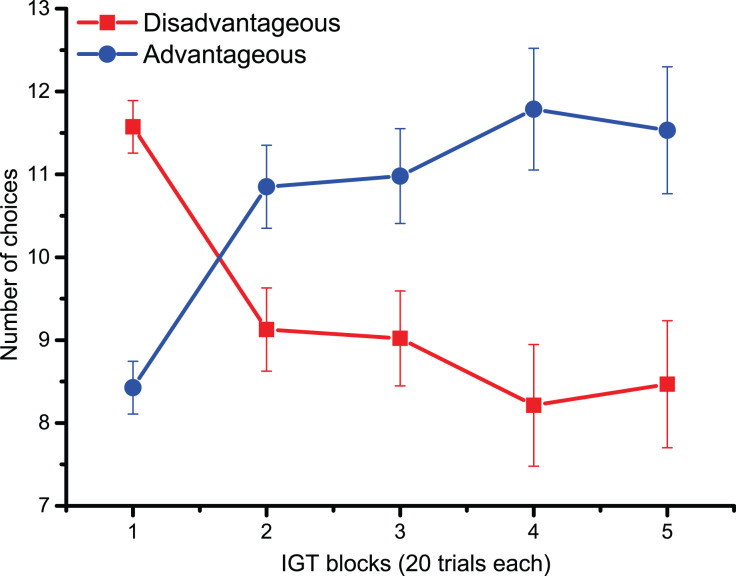
Behavioral data. IGT learning trend (*i.e*., the number of choices of advantageous/disadvantageous decks) across the five blocks. Participants gradually learnt to favor advantageous over disadvantageous decks.

For behavioral data results, as shown in Fig. 2, the statistically significant two-way interaction between Block and Deck Type [*F*_(2.9,133.5)_ = 9.5, *p* < 0.001, η_p_^2^ = 0.17] indicates the typical learning trend seen during the IGT. Pairwise comparisons revealed that during the first 20 trials (Block 1) participants chose disadvantageous decks more often (*p* < 0.0001). However, starting from Block 3 (trials 40–60), advantageous decks were preferred (all pairwise comparisons < 0.05), although confidence intervals revealed variability in the participants’ choices. The lack of significant main and interaction effects for Cognitive Inflexibility indicated that it had no impact on the participants’ deck type preference or learning over successive blocks [*F*_(1,43)_ = 0.370, *p* = 0.546].

For cardiac data, there was a main effect of Monetary Feedback (post-choice information on Losses/Wins) [F _(1,44)_ = 26.4, *p* < 0.0001, η_p_^2^ = 0.37], indicating larger cardiac accelerations after wins compared to losses. A significant interaction between Monetary Feedback and Bins was found across trials [F _(2.7,119.3)_ = 12.1, *p* < 0.0001, η_p_^2^ = 0.22]; pairwise comparisons revealed that 1.5 s after the feedback (from Bin 3), HR was larger following monetary gains compared to losses (pairwise comparisons < 0.01 after 1.5 s; all comparisons < 0.0001 2.5–5.5 s after monetary feedback). The significant main effect of Cognitive Inflexibility [F _(1,44)_ = 5.2, *p* < 0.05, η_p_^2^ = 0.11] indicated that cognitively flexible participants had a higher heart rate, regardless of monetary feedback; no further effects reached statistical significance.

**Figure 3:**
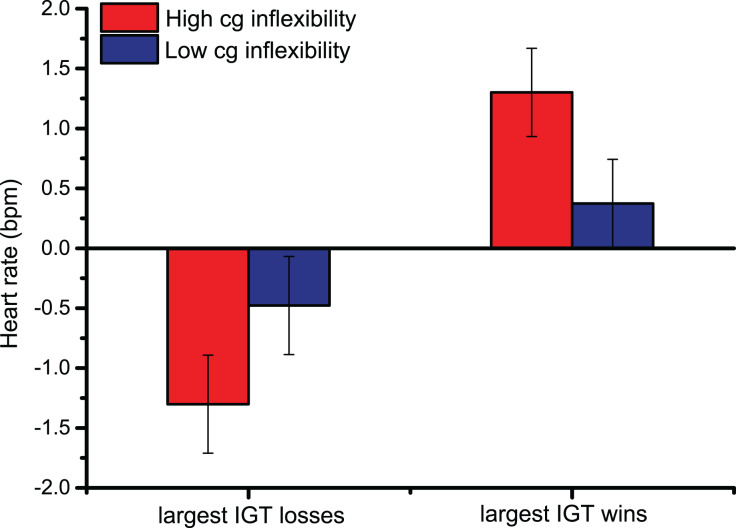
Cardiac data after the Largest Monetary Feedback. Group differences in heart rate (bpm) were found following the largest monetary gains, suggesting greater sensitivity to rewards in cognitively inflexible participants.

In heart rate changes after the Largest Losses/Wins, the main effect of Largest Monetary Feedback [*F*_(1,44)_ = 20.9, *p* < 0.001, η_p_^2^ = 0.32] indicated that the upper 10^th^ percentile of monetary gains prompted larger cardiac accelerations than the greatest losses, confirming the pattern observed following overall Losses/Wins feedback. Similarly, pairwise comparisons of the significant interaction between Largest Monetary Feedback and Bins across trials [*F*_(2.3,99.2)_ = 8.4, *p* < 0.001, η_p_^2^ = 0.16] showed that, 2 s after feedback was provided, HR changes were larger, indicating cardiac accelerations, following the greatest monetary gains (the pairwise comparisons were < 0.01 2 s after the largest Losses/Wins; starting from 3 s after the start of the trial until the end of the trial, all *p*-values were < 0.001). The significant interaction between Cognitive Inflexibility and the Largest Monetary Feedback [*F*_(1,44)_ = 5.4, *p* < 0.05, η_p_^2^ = 0.11], in Fig. 3, indicated that only cognitively inflexible participants showed larger cardiac accelerations after monetary gains, reflecting greater sensitivity to rewards (*p* < 0.001); this was not seen in low cognitive inflexibility individuals.

## Discussion

The present study examined whether nonclinical individuals with high cognitive inflexibility show poor performance and physiological hyper-reactivity to rewards during a complex decision-making task. The data indicated that, over time, the participants learned to avoid disadvantageous options, thus confirming the behavioral patterns expected in the IGT. However, cognitive inflexibility did not affect performance. When we examined sensitivity to rewards (as indexed by cardiac reactions to IGT wins), we observed that, in line with the literature, monetary gains prompted greater cardiac accelerations than monetary losses (Goudriaan et al., 2006; Miu, Heilman & Houser, 2008; Studer, Scheibehenne & Clark, 2016). Moreover, when comparing reactions to the largest monetary wins and losses, we observed that only cognitively inflexible individuals showed larger cardiac accelerations during the greatest wins. Taken together, the data confirmed the hypothesized role of individual traits in modulating reactions to rewarding cues, where individuals with high cognitive inflexibility showed greater reward sensitivity. However, cognitive inflexibility did not modulate performance on the IGT, as evidenced by the number of disadvantageous choices, therefore indicating a mismatch between behavioral performance and post-choice physiology (which is discussed below).

Cognitive inflexibility, which manifests behaviorally as difficulty switching to an alternative option when a previously rewarding cue leads to negative outcomes, has been identified as a core feature of psychiatric disorders characterized by compulsivity, such as behavioral and substance use disorders (Banca, Harrison & Voon, 2016; Perandrés-Gómez et al., 2021). Recent theories on the development of addictions assign a central role to cognitive inflexibility as a transdiagnostic impairment of higher-order cortical functions (*e.g*., Parnaudeau, Bolkan & Kellendonk, 2018; Voon et al., 2017) that accompanies compulsive behavior, and might act both as a preexisting trait and a drug-induced deficit (Groman et al., 2019). According to these theories, cognitive flexibility is expected to aid decision-making by promoting efficient alternation between reward-driven (“model-free”) and long-term (“model-based”) strategies (Groman et al., 2019; Voon et al., 2017; Wyckmans et al., 2019). Although this dichotomy has been criticized as too simplistic and narrow given the heterogeneity of decisional strategies (Collins & Cockburn, 2020; Deserno & Hauser, 2020; Gueguen, Schweitzer & Konova, 2021; Vandaele & Ahmed, 2021), both clinical and animal data indicate that individual differences in reward sensitivity play a key role in decisional strategies adopted during pathological reinforcement learning (Fraser & Janak, 2019; Groman et al., 2019). Consistent with this, the nonclinical university students in the present study scoring highly for cognitive inflexibility showed greater cardiac accelerations after the largest wins compared to cognitively flexible participants. Here, it should be noted that greater reward sensitivity has repeatedly been observed in psychiatric and substance use disorders characterized by cognitive inflexibility (Groman et al., 2019; Parnaudeau, Bolkan & Kellendonk, 2018; Perandrés-Gómez et al., 2021; Verdejo-García, Lawrence & Clark, 2008; Wyckmans et al., 2019). By contrast, the current study found an association between higher cognitive inflexibility and heightened reward sensitivity in nonclinical individuals, thus confirming an association between a psychological trait (cognitive inflexibility) and physiological correlate (cardiac hyper-reactivity to rewards) in a sample free from psychopathology.

Concerning the role of sensitivity to reward in the context of cognitive inflexibility, an fMRI study (Gusnard et al., 2003) investigated neural correlates of cognitive inflexibility, as assessed by Cloninger’s TCI Persistence Scale (Cloninger et al., 1994). Cognitive inflexibility was consistently associated with greater (high-cognitive inflexibility individuals) or reduced (low-cognitive-inflexibility individuals) activity in areas of a neurocircuit including the occipital and medial prefrontal cortices and ventral striatum; this circuit is functionally associated with “reward-related activities” and hypothesized to “guide behavior based on contextually relevant incentive-based information” (Gusnard et al., 2003). The current data therefore confirm Gusnard and colleagues’ findings, suggesting that cognitive inflexibility might be associated with greater reward sensitivity, as indexed by central and peripheral physiological markers.

In the IGT, it has been repeatedly observed that monetary gains prompt cardiac accelerations (Crone et al., 2004; Goudriaan et al., 2006). In tasks related or unrelated to gambling, increased heart rate reliably accompanies the presentation of rewarding, pleasant cues to such an extent that cardiac accelerations during emotionally relevant cues are regarded as a reliable index of appetitive motivation, *i.e*., the evolutionarily adaptive urge to pay sustained attention to motivationally positive cues (Anderson & Brown, 1984; Bradley, Cuthbert & Lang, 1993; Bradley et al., 2001; Greenwald, Cook & Lang, 1989). However, in the Iowa Gambling Task, the processes through which sensitivity to rewards may influence performance are less clear. Bechara & Damasio (2000) hypothesized that, by increasing monetary gains, participants would be more motivated and thus perform better. However, the data indicated that performance did not improve with larger wins (ibidem; Lee et al., 2014). Similarly, in the frequently observed ‘prominent deck B’ phenomenon (Crone et al., 2004; Lin et al., 2007; Visagan, Xiang & Lamar, 2012), a high frequency of wins associated with deck B makes it difficult for participants to resist it despite ‘its bad final outcomes’ (see Visagan, Xiang & Lamar, 2012). Based on the current findings, we hypothesize that high monetary wins and the associated cardiac accelerations might create an overall feeling of pleasure wherein some IGT cues assume greater motivational relevance; this rewarding context might distract some vulnerable individuals from long-term, gradual gains and bias them toward immediate ones that are in fact best avoided. The data presented here therefore seem consistent also with the literature on delayed reward discounting, which indicates that there is a decrease in subjective value of any reward that must be postponed: it is difficult to refrain from selecting immediate rewards (Bari & Robbins, 2013; Bickel et al., 2014; Hamilton & Potenza, 2012; Stevens et al., 2014). As an individual trait, delay discounting has been ‘linked to unfavorable addiction treatment outcomes’ (Stevens et al., 2014) and identified as an endophenotype for multiple psychiatric disorders (Bickel et al., 2019). Further data are needed to elucidate the behavioral and physiological correlates of the traits that render some individuals with high reward sensitivity vulnerable to poor decision-making.

In the current study, the association between subjective cognitive inflexibility and physiological reward sensitivity was not accompanied by worse performance. In line with previous studies (*e.g*., Crone et al., 2004; Rivalan, Ahmed & Dellu-Hagedorn, 2009; Werner, Duschek & Schandry, 2009), the discrepancy between performance and post-choice physiology seen herein suggests that trial-by-trial rewards take precedence over long-term strategies (Crone et al., 2004). Consistent with this, it has been argued that pathological hypersensitivity to reward might impair decision-making because of biases in motivational relevance rather than failure to acquire relevant information (Rivalan, Ahmed & Dellu-Hagedorn, 2009). The present data confirm that exaggerated reward sensitivity, as indexed by physiological reactivity, can occur despite adequate information processing, as indexed by performance in the IGT. The observation of discrepancies among self-report, behavioral, and physiological data is not uncommon in emotion research: among fear disorder patients, those who report the most severe symptomatology tend to show reduced physiological reactivity to phobic cues (Lang, McTeague & Bradley, 2016; McTeague & Lang, 2012). Similarly, among nonclinical participants presented with emotionally relevant cues, subjective and behavioral indices frequently diverge from physiological responses (Bradley et al., 2001; Bradley & Lang, 2007). Therefore, the current findings are consistent with research and clinical perspectives, and demonstrate the importance of combining physiological, behavioral, and self-report indices of psychopathology to provide clinicians and researchers with more complete and reliable reference data (Insel & Cuthbert, 2015); the ultimate goal of this approach is to identify vulnerable individuals with respect to a wide spectrum of psychopathologies, and to tailor treatment based on specific biobehavioral correlates (Kypriotakis, Cinciripini & Versace, 2020).

The current findings might help account for previous inconsistencies regarding the role of cognitive inflexibility in addictions: while some data indicate that cognitive inflexibility assists individuals in refraining from maladaptive habits, other data show that its presence constitutes an obstacle to recovery (*e.g*., Etter, 2010; Kalman et al., 2010; López-Torrecillas et al., 2014). We hypothesize that such discrepancies might originate from a lack of conceptual clarity, given that cognitive (in)flexibility has been regarded as a negative trait reflecting cognitive rigidity and resistance to change (*e.g*., Abbate-Daga et al., 2014; López-Torrecillas et al., 2014; Perandrés-Gómez et al., 2021), as well as a positive trait akin to endurance (*i.e*., ‘grit’ in the face of adversity; Cloninger et al., 1994; Etter, 2010; Kalman et al., 2010). Indeed, the more recent TCI-R takes a wider view of the construct of cognitive inflexibility, such that it can have both positive and negative outcomes (Josefsson et al., 2013). The data reported here suggest that sensitivity to rewards might predispose cognitively inflexible individuals to suboptimal decision-making. Thus, the findings imply that cognitive inflexibility hinders rather than assists individuals in terms of abstaining from maladaptive habits. The generalizability of the results, however, would be improved by studies refining the concept of cognitive inflexibility, and subsequently validating it.

## Conclusions

In summary, cognitive inflexibility and sensitivity to rewards are common in compulsive disorders. We examined whether, in a nonclinical sample, cognitive inflexibility was similarly associated with worse decision-making or increased appetitive reactions to wins. Cognitive inflexibility did not alter performance; however, cognitively inflexible participants showed large cardiac accelerations during the largest wins. Cardiac accelerations, as a reliable biological marker of appetitive motivation, suggest that greater sensitivity to rewards might render cognitively inflexible individuals more vulnerable to the largest gains, thereby predisposing them to inadequate decision-making. The findings, in line with recent research and clinical perspectives, demonstrate the relevance of incorporating physiological, behavioral, and self-report markers/indices of psychopathology, to identify vulnerable individuals and tailor treatment based on specific biobehavioral correlates.

## Supplemental Information

10.7717/peerj.15318/supp-1 Supplemental Information 1 Raw Data for Figure 1.

10.7717/peerj.15318/supp-2 Supplemental Information 2 Raw Data for Figure 2.

## Additional Information and Declarations

### Data Availability

The following information was supplied regarding data availability: The raw data are available in the Supplemental Files.
